# ‘Flower of the body’: menstrual experiences and needs of young adolescent women with cerebral palsy in Bangladesh, and their mothers providing menstrual support

**DOI:** 10.1186/s12905-020-01032-3

**Published:** 2020-08-01

**Authors:** R. Power, K. Wiley, M. Muhit, E. Heanoy, T. Karim, N. Badawi, G. Khandaker

**Affiliations:** 1 Discipline of Child and Adolescent Health, Sydney Medical School, University of Sydney, Cnr Hawkesbury Rd and Hainsworth St, Locked Bag 4001, Westmead, NSW 2145 Australia; 2 Asian Institute of Disability and Development (AIDD), University of South Asia, Dhaka, Bangladesh; 3 Sydney School of Public Health, Faculty of Medicine and Health, University of Sydney, Sydney, NSW Australia; 4 CSF Global, Dhaka, Bangladesh; 5 Cerebral Palsy Alliance Research Institute, University of Sydney, Sydney, NSW Australia; 6 Central Queensland Public Health Unit, Central Queensland Hospital and Health Service, Rockhampton, QLD Australia

**Keywords:** Cerebral palsy, Disability, Menstruation, Reproductive and sexual health, Bangladesh, Global South, Low- and middle-income country, Adolescent, Teenager

## Abstract

**Background:**

This study offers voice to young adolescent women with cerebral palsy (CP) in Bangladesh as they describe their menstrual experiences and needs, and their mothers providing menstrual support.

**Method:**

Semi-structured focus groups with adolescents with CP, and separately their mother. Data was analysed using a material discursive framework and drawing on feminist disability theory. Participants were recruited from the Bangladesh CP Register (BCPR); a population-based surveillance of children and adolescents with CP in rural Bangladesh.

**Results:**

Participants were 45 women including 12 female adolescents with CP and 33 female caregivers. Participants reported a wide range of experiences and needs; menarche acted as a gateway to menstrual information although for some a discourse of silence prevailed due to exclusion from peer and familial networks. Menstruation was discursively constructed as a sign of ‘female maturation’ marked by an expectation of ‘independence’, required for acceptance into socially valued adult roles, and was positioned alongside increased vulnerability to sexual abuse. Young adolescent women with CP were expected to ‘quietly endure’ the material aspects of menstruation although unmanaged pain and distress were described. Mothers reported an imperative for meeting their adolescent’s menstrual needs however this role was discursively positioned as ‘painful’, ‘irritating’ and ‘shameful’, in part due to an absence of affordable, functional menstrual resources.

**Conclusion:**

The findings of the present study provide motivation for disability services in Bangladesh to account for the menstrual needs of young adolescent women with CP within service delivery through strategies such as providing menstrual education and by embedding value in constructs such as ‘interdependence’. Moreover, interventions focused on alleviating menstrual pain among young adolescent women with CP as well as those targeted to alleviate distress among mothers providing menstrual care are required. Finally, policy responses are required to ensure that ‘inclusive development’ considers the needs of menstruating women with disability.

## Background

Menstruation is often an important marker in a young woman’s life, the meaning and significance of which will be shaped by a woman’s personal, cultural and political context [18]. For girls and young adolescent women with cerebral palsy (CP) in rural Bangladesh menstruation may hold unique significance and require specific responses, as women navigate their material bodies alongside complex beliefs and stigmas surrounding both disability and menstruation in a low economic rural setting. The menstrual experiences and needs of girls and young adolescent women with CP are rarely accounted for in menstrual discourse, particularly in low- and middle-income countries (LMICs) [8, 13].

CP is one of the leading causes of childhood physical disability and refers to a group of disorders causing impairments to a person’s movement and posture [24]. Bangladesh, a typical LMIC, estimates 3.4 per 1000 children to have CP [19]. More than two thirds of these children require wheeled mobility and more than half have cognitive or speech impairments [19]. Although mortality of children with CP in Bangladesh is high [16] more children than ever before are surviving into adulthood; understanding menstrual experiences and needs is thus pertinent.

Previous research suggests that menstruation is discursively positioned as ‘impure’ and ‘dirty’ in Bangladeshi society [30] portraying females as ‘inferior’ and establishing a foundation for life-long disempowerment [8]. Girls and young adolescent women are likely to be ‘uninformed’ and ‘unprepared’ for menarche due to incomplete and inaccurate knowledge about menstrual physiology and hygiene and lack of access to affordable hygienic menstrual products and water, sanitation and hygiene (WASH) facilities (i.e. private accessible latrines) [8]. Moreover, numerous studies report disruption to education for young women during menstruation, resulting in long term economic disadvantage and contributing to women’s oppression [1, 14]. Some anecdotal evidence however documents the resistance of young women to menstrual oppression instead celebrating and advocating for menstrual issues.

For girls and young adolescent women with CP in Bangladesh it is reasonable to expect that some aspects of menstruation will differ [17]. To date however, literature about the menstruation of females with CP has predominately adopted a biomedical approach and focused on topics such as menstrual issues [33], gynaecologic complaints [7], contraceptive prescription [10] and have been conducted almost exclusively in the global north. This paper, focused in Bangladesh, adopts an understanding of menstruation as a naturally occurring process that is biological as it is social and cultural, and draws on a feminist disability perspective amplifying the often absent voices of girls and young adolescent women with disability, and their mothers. Our approach understands menstrual hygiene management to be a universal and intersectional issue, attainment of which is required to achieve more than a third of the sustainable development goals (SDG’s) [28] and is essential for the equity of girls and women. Thus, our research questions are (a) how do young adolescent women with CP in Bangladesh discursively construct their menstrual experiences and needs, and (b) what are the experiences and meanings applied by their mothers in providing menstrual support.

## Method

### Study design

This study is part of a broader mixed-methods project focused on the wellbeing of adolescents with CP in rural Bangladesh. The project has previously examined adolescents’ health-related quality of life (HRQoL) and mental health. In this paper we present data from the qualitative stage of the project interested in reproductive and sexual wellbeing; we focus specifically on the experiences of young adolescent women with CP regarding menstruation and their mothers in providing menstrual support.

### Participants

All young adolescent women clinically diagnosed with CP, aged 10 to ≤18 years (a normative classification of adolescence in Bangladesh) [25], were identified from the Bangladesh Cerebral Palsy Register (BCPR) and invited to take part. BCPR is the first population-based register holding data on the socio-demographic and clinical characteristics of children and adolescents with CP in a LMIC and covers a defined geographical region of the Shahjadpur sub-district of Sirajganj district in the northern part of Bangladesh. Details of BCPR are described in Khandaker et al. [20].

We also requested participation from each adolescent’s primary female caregiver classified as a mother, grandmother, aunt, sister or other female relative who provided majority of care and support. We applied principles of maximum variation sampling to our recruitment methods, to ensure representation of participants across a range of ages, motor functioning (i.e. severity), intellectual capacity and socioeconomic status.

### Procedure

Participants were contacted by phone and invited to participate in a focus group. Due to high rates of illiteracy in the study area, participants were verbally provided with information about the nature of the interview and list of interview topics. To ensure understanding and voluntary informed consent, this information was provided both at the time of invitation, and again on arrival for the interview. Participants were also given a phone number for contact with a research officer that they could call to ask questions about the interview.

Prior to the interview, informed verbal and written consent was obtained for all individual participants. In cases of illiteracy, written consent was obtained by thumbprint. Minors (i.e. <16y) provided verbal assent and their parent or legal guardian written consent. In cases where adolescents were unable to consent (i.e. severe communication impairment or perceived lack of capacity) then consent was only obtained from the parent or legal guardian and data was only collected via the caregiver. No data was collected in instances that adolescents indicated objection to participation, even in instances of parental consent.

Semi-structured focus group interviews were conducted with small groups of female adolescents with CP and separately small groups of their female caregivers. The interview schedules were initially piloted with two groups to ensure feasibility. The interview schedules were then refined to incorporate local dialect and improve in-depth probing. The adolescent interview schedule explored adolescents’ experiences, attitudes and beliefs about topics such as puberty, menstruation, erections, masturbation, relationships, marriage, sexual abuse and sources of information and support. The present study reports only on data related to menstruation. The caregiver interview schedule covered the same topics; caregivers were asked to answer ‘about’ their adolescent (i.e. as proxy report) and were also asked about their roles providing menstrual care.

Interviews were conducted in Bengali by a female researcher for whom Bengali was her day-to-day language, and were audio recorded. Participants received a small financial reimbursement for expenses incurred as a result of their participation.

Observational data was recorded in journal format during the interviews and reflexive notes made throughout the research process by the first author. This allowed for reflection by the author to understand their subjective positioning in relation to participants experiences and discourse.

This study adhered to COREQ guidelines and has ethical approval from the Bangladesh Medical Research Council (BMRC/NREC/2013–2016/1165) and University of Sydney Human Research Ethics Committee (2016/646). All procedures performed in this study were in accordance with the ethical standards of these institutes and with the 1964 Helsinki declaration and its later amendments or comparable ethical standards.

### Analysis

Interviews were transcribed verbatim in Bengali. The Bengali transcripts were then translated to English by a speaker fluent in both Bengali and English but for whom Bengali was their day-to-day language. The translator was given instruction to focus on conceptual equivalence rather than literal word for word translation and to remove identifying information from the transcripts. The translated (English) transcripts were then read by members of the research team fluent in both languages to confirm acceptability of the translations and integrity to the original recording; conceptual discrepancies between the Bangla and English versions were resolved. English speaking members of the research team then read the final English transcripts to confirm understanding.

Adolescent and maternal data were initially analysed separately. The transcripts were read in an ‘active way’ [6] to identify patterns across the data and coding frames developed using a hybrid of deductive and inductive methods. Data was coded using NVivo 11 and thematically analysed using a material-discursive approach (see [29, 32]) and drawing on feminist disability theory (see [11]). Our approach acknowledges that the materiality of menstruation (i.e. the physical aspects of the experience) is inseparably interested in menstrual discourse (i.e. the meaning ascribed to menstruation in relation to influences from an individual’s life, cultural and political context). Both meaning and materiality are considered mutually necessary for understanding to be attained [5].

## Results

Five themes were identified during our thematic analysis; (1) Menarche as a gateway to information; (2) Menstruation as a sign of female maturation – new expectations and exclusions; (3) Independence and support needs (4) Menstrual symptoms and an imperative to endure quietly and (5) Menstrual regulation. These are discussed following an overview about the study participants.

### Participants

Three focus group interviews were conducted with adolescents with CP in groups of 3 to 6 participants, and 9 focus group interviews with female caregivers of adolescents with CP in groups of 4 to 7 participants. Focus group interviews ranged between 30 and 62 min in length; longer focus groups tended to be those with higher numbers of participants.

In total, participants were 12 young adolescent women with CP and 33 female caregivers. Of the caregivers, 21 reported on behalf of adolescents who were unable to self-report. Caregivers were mothers (*n* = 30, 90%), a grandmother (*n* = 1, 3.3%), a sister (*n* = 1, 3.3%) and an aunt (*n* = 1, 3.3%). Throughout the remainder of present publication, caregivers are referred to as mothers unless a specific relationship to the adolescent with CP is identified.

Socio-demographic characteristics of participants are detailed in Table 1. Majority lived in kutcha (impermanent mud houses) or semi-pucca (semi-permanent) housing and lacked access to clean water. Moreover, only a minority of adolescents and their mothers reported educational attainment.

**Table 1:** Sociodemographic characteristics of adolescents with CP by reporting method (i.e. self- or proxy-report)

Socio-demographic characteristics	Self-report (*n* = 12)	Proxy-report only (*n* = 21)
Age of adolescents with CP (mean years, SD)	14.5 (1.8)	15.0 (1.8)
GMFCS (*n*, %)		
I	2 (16.7%)	4 (19.0%)
II	5 (41.7%)	4 (19.0%)
III	3 (25.0%)	2 (9.5%)
IV	1 (8.3%)	4 (19.0%)
V	1 (8.3%)	7 (33.3%)
Associated impairments (*n*, %)		
Epilepsy	4 (33.3%)	6 (28.6%)
Intellectual impairment	4 (33.3%)	14 (66.7%)
Visual impairment	0 (0%)	2 (9.5%)
Hearing impairment	0 (0%)	5 (23.8%)
Speech impairment	5 (41.7%)	17 (81.0%)
Attends school (*n*, %)	7 (58.3%)	3 (14.3%)
Mother primary or above education	6 (50.0%)	5 (23.8%)
Monthly family income (median, IQR)		
BDT	7500 (4750)	6000 (3000)
USD	88 (56)	71 (35)
Type of housing		
Kutcha	4 (33.3%)	16 (76.2%)
Semi-pucca	7 (58.3%)	0 (0.0%)
Pucca	2 (16.7%)	4 (19.0%)
Source of drinking water - clean tap water ^a^	0 (0.0%)	2 (9.5%)
Sanitary latrine ^b^	7 (58.3%)	17 (81.0%)

*GMFCS* Gross Motor Function and Classification System, *BDT* Bangladeshi Taka, *SD* Standard Deviation, *IQR* Interquartile Range

^a^Remainder = tube well water

^b^Remainder = no toilet facility or non-sanitary latrine

Menarche had occurred for all the young adolescent women with CP except two. Of those two young women, one knew about it, but said it hadn’t commenced, and the other stated that she didn’t know about it, ‘I don’t know what it is. My grandma doesn’t tell me’. These two young women remained present for discussion about menstruation, and at times contributed, however were not asked direct questions. Majority of the young women used cloth napkins made from old clothes during menstruation although one relied on toilet paper. Single use sanitary pads/ napkins were rare with only one participant having access to these in instances such as ‘If I have to go somewhere or if my cloth napkin gets spoilt.’

## Menarche as a gateway to information: ‘We can talk about [menstruation] with those who have it’

Common among young adolescent women with CP, was a discourse of silence about menstruation. When it was discussed, menstrual knowledge was typically obtained through informal conversation and reserved for sharing between those with a similar experience. Menarche acted as the gateway to this information, adolescents with CP explained *‘we can talk about [menstruation] with those who have it’* such as ‘with our friends’, ‘sister-in-law’ and ‘grandmother’. Mothers were often ‘off limits’ for this topic of discussion, assuming positions regulating personal disclosures and enforcing social norms restricting discussion of menstruation, as was the case with this mother:

*‘Nowadays, she comes and tells me what has happened to her. Then I have to explain that these changes occur when one grows up but that these private details are not meant to be said aloud.’*

Some girls and young adolescent women with CP resisted this notion, stating that they *would* talk to their mothers as *‘mothers are our friends’* and identifying their mothers as their primary source of information and support. Others however were entirely excluded from menstrual discourse and learning about menstrual etiquette due to lacking peer networks, rejection from relatives, and assumed incapacity to comprehend, *‘she cannot understand anything’* and *‘my daughter’s [period] has started but she can’t tell … she cannot do anything, there is no point’*.

For those young adolescent women with CP for whom menstrual information was available, teaching tended to focus almost exclusively on the periodicity of menstruation, for example:

*‘After her [my daughter’s] first period, when she had her second period, she understood that she gets periods. My sister-in-law explained it to her. After that she understood that she will get her period every month.’*

## Menstruation as a sign of female maturation – new expectation and exclusions: ‘Flower of the body’

Menstruation was discursively constructed as a sign ‘that the girl is mature’, illustrated with the phrase ‘flower of the body’. For some young adolescent women with CP this signified increased independence and the opportunity to assume new responsibilities such as ‘cleaning up’, ‘dusting’ and ‘cooking’.

For other young women with CP, notably those with higher support needs, this stage of development was interpreted by mothers to reflect physical growth only, for example:

*‘My daughter is just growing physically; there is no development of cognitive ability or senses’*

The concept of ‘maturity’ represented complex expectations of, but potential exclusion from, socially valued roles such as marriage and childbearing. Young adolescent women with CP tended to be unclear about their capacities to engage in these roles. One young adolescent woman with CP stated, *‘Disabled people should get married but they can’t consummate their marriage’.* Another, when asked if women with CP could have children replied, *‘That I don’t know’*. Mothers on the other hand, understood their children’s fertility *‘It seems like she will give birth if she is married off’* and this was positioned alongside increased vulnerability to sexual abuse. Mothers often needed to negotiate the risks of leaving their child at home in order to attend to work and familial duties, and described feeling ‘worried’, ‘anxious’ and ‘scared’:

*‘I wish I could stop my daughter’s growth. A disabled person does not have the strength to defend herself. What if we go somewhere leaving our daughter alone at home and then somebody rapes her and gets her pregnant … ? It’s not possible to take her everywhere. I feel anxious thinking about my daughter when I’m outside.’*

## Independence and support needs: ‘I clean it myself’ and ‘Now that she is menstruating, I have to help her with that also’

Taking responsibility for the timing of menstruation and changing and cleaning one’s own menstrual cloths appeared to be important for young adolescent women with CP in positioning oneself as adult. Some young adolescent women with CP (predominantly those who participated in the focus group) managed their menstrual needs either independently or through a negotiated inter-dependence. One young adolescent woman with CP proudly explained, *‘I use cloth during menstruation. I clean it by myself after using it.’*

For others, significant support was required. In these instances mothers described an imperative for meeting their adolescent’s needs, as one mother stated, *‘If I don’t take care of her then nobody else will’.* Involvement in the provision of menstrual care was described as ‘painful’, ‘irritating’ and ‘shameful’, and presented challenges, for example one mother stated:

*‘This change is very painful for me. I have to keep her pubic washed and cleaned every 4-5 days. … I have no shame for I have to do this work? But Allah has given her to us and so I have to do this. … I cannot take her to different places [while she is menstruating], she cries but I cannot take her as she cannot keep her cloth in one place. … If she moves then everything spreads all over the place. I place a weight on her and from time to time I change [the menstrual] cloth.’*

Mothers also described difficulties in providing care as their daughters became older:

*‘It is very difficult for me to carry her and give her a shower now [that she is this age]. There is no one to hold her without me. I cannot give her a shower by myself.’*

Although many described needing help, mothers often had to negotiate these tasks without exposing the events to other household members including the adolescent’s male siblings and father. This negotiation was described by a mother whose daughter required full support with menstrual care:

*‘I basically close the door of my house. Then I clean everything and open the door. I do not let them [the men in the house] know.’*

## Menstrual symptoms and an imperative to endure quietly: ‘I tell her that she has to bear with it and Allah has given this’

Young adolescent women with CP described a range of menstrual symptoms, attributed to the material body with accounts including *‘My hands and legs pain. I feel dizzy. My stomach aches’*, *‘my waist hurts’* and *‘my back hurts a lot’*. Some young adolescent women with CP, described symptoms as minor, lasting only a day or two, *‘It pains in the first day only, other than that, it doesn’t’.* These participants reported minimal impact on day-to-day activities. Three young women with CP said they attend to their normal chores during menstruation, *‘we do everything’* although one reported missing prayer, *‘Sometimes it starts all of a sudden when I go inside the toilet. I skip offering prayers then’*.

Mothers also described their perceptions of their daughter’s symptoms *‘her head goes blank, she doesn’t eat, she becomes weak and feels giddy’* and *‘she gets convulsions every now and then. It escalates during menstruation’.* Concerns about menstrual problems were reported by a few mothers including one whose daughter had been *‘menstruating since last the 15 days’* and another who stated:

*‘She lies on her back and urinates and defecates. It smells very bad. I clean everything with powder, soap. It takes longer to feed her. She drinks water little by little. She gets constipation, white discharge, after that she gets periods. Period lasts for 2-3 days for most people, but she has it for seven days.’*

Throughout these accounts, menstruation and the ensuing symptoms were positioned as something ‘given by Allah’ and to be quietly endured, a responsibility for female caregivers to enforce but driven by paternal forces:

*‘I tell her it is a matter of shame. Your brother or your father is coming and what will they say if they see all this? It will get better and will be gone. Do not make all this noise. Allah has given this and everyone has this.’*

For some young adolescent women with CP however menstruation appeared to be very distressing, and was not quietly endured, *‘She screams when she has her period, she is afraid’* and *‘when my daughter has monthly periods she cries loudly’*.

## Menstrual regulation: ‘The doctor forbade me to give her any medicine’

When the young adolescent women with CP were asked about taking medication, either to manage pain or for menstrual regulation (i.e. contraception), majority replied that they didn’t understand. The following information is provided by mothers.

Medications associated with menstruation were rarely used by young adolescent women with CP although mothers considered their potential use in instances that the young women couldn’t self-regulate pain and/or distress:

*‘My daughter screamed for 3-4 days when she started menstruating. Then I bought homeopathic medicines for her. Now I let her take paracetamols. It reduces her stomach ache. I also give her sedatives. I can’t control her. She troubles me a lot.’*

Although providing menstrual care was a strain for majority of the mothers, menstrual bleeding was discursively positioned as healthy, *‘it’s good for the health if the menstrual blood gets cleared out’*. For this reason, although some mothers were interested in contraception, *‘it’s better to stop it if it isn’t harmful to their health’*, majority were concerned about potential health effects, and for this reason would not consider contraceptive use with their daughters. One mother explained:

*‘Our mothers and elders have told us that if the period stops quickly, then it may be harmful for your body … There are people who say that one can lose vision in an early age. If the blood breaks, it can be good for your health and movement … if you take medication to prevent it, then it might be harmful in other ways.’*

Fathers and healthcare providers assumed positions of power and enforced these views. A couple of mothers had made enquiries about menstrual regulation for their daughters however, were forbidden from doing so *‘I consulted a Doctor after my daughter started having her periods. The Doctor forbade me to do anything like that’* and *‘My husband talked about that matter. They forbade us to do that. It’ll be harmful for her’.*

## Discussion

Young adolescent women with CP in rural Bangladesh reported a complex picture of learning about and navigating the materiality of menstruation alongside restrictive discourses of ‘maturation’, and ‘quiet endurance’. Our findings offer insight into how these notions are negotiated and have practical implications for policy, research and service providers working with girls and young adolescent women with CP and their mothers in Bangladesh.

That a discourse of silence exists around menstruation and that there is a requirement for menarche to access information is perhaps not surprising. However, what is interesting are our findings of resistance to these ‘norms’ as young adolescent women with CP and their mothers adjust to meet each other’s needs and expectations in this area. Menses can result in complex negotiations for young women with CP as they forge their social positions as young adults whilst negotiating the assumptions and judgements of others in inherently ableist contexts. Adolescents in the present study relied heavily on maternal support with menstruation, placing them in contrast to their peers without disability who although may ‘lack confidence’ in some aspects of menstrual management, are likely to manage their hygiene needs independently and discretely [15, 34]. This contrast in maternal involvement may disrupt narrowly defined expectations of ‘maturation’ and ‘independence’ for young adolescent women with CP. An ideological shift away from the laden dichotomy of dependent versus independent towards the valuing of interdependence and challenging the often inherent assumption that independence is equated with adult maturity may help to positively position young women as active agents in the negotiation of their menstrual care needs and subjects of power, rather than pity [22].

More than half of participants in the present study reported intellectual impairment; previous research indicates that with support women with intellectual impairment(s) and/or high support needs can understand and accept their menstruation [2, 23] however, this is best achieved via the provision of menstrual education in the years leading up to menarche allowing time for knowledge acquisition and practice [12], and if the teaching tools and approaches are adapted to meet participants specific learning needs [31]. Menstrual interventions in Bangladesh for young adolescent women with CP and their mothers need to address these considerations and the conflicting requirement of menarche as a pathway to knowledge, as well as be physically, financially and socially accessible and inclusive.

Our findings provide new understanding of the complex, paradoxical narratives surrounding menstrual caregiving. Mothers held almost exclusive responsibility for this role however appeared to be subjugated in decision making by patrilineal, patriarchal regulators and, alongside their daughters, required to ‘endure quietly’. Maternal discourse juxtaposed the materiality of menstruation as ‘unclean’ against the recursive nature of menstruation, constructed as ‘healthy’ and menstrual caregiving as burdensome and shameful for mothers, but non-negotiable. Maternal concerns were positioned as secondary to daughter’s health, demonstrated in discourse about menstrual regulation. Bangladeshi mothers aren’t alone, their accounts were similar to Indian mothers of adolescents with disability [26] although in a Taiwanese study [9] mothers did not perceive menstrual caregiving as a difficult task, likening it to their own menstrual needs, and constructing it as ‘healthy’. The Taiwanese mothers drew on cultural understandings of ‘fate’ as a coping strategy. Future research may offer insight into how mothers bring their own experiences of menstruation to menstrual caregiving and how disability and public health systems may acknowledge and respond to these realities.

Improved access (i.e. availability and cost) to resources for menstrual hygiene management such as absorbent (reusable) menstrual pads that can be kept securely in place and that meet the functional needs of young women who are sitting or lying for long periods, and for those with heavy flow, as well as water, sanitation and hygiene (WASH) solutions that are oriented to the needs of menstruating females with disability have potential to improve the hygiene and physical health outcomes of young women with CP. Urinary tract infections (UTI’s), scabies in the vaginal area, and abnormal abdominal pain are common issues linked to inadequate menstrual hygiene [27].

Finally, dysmenorrhea is believed to affect more than 70% of young women [3, 4] and can contribute to behavioural problems and distress for those who cannot communicate their experiences [21]. Understanding and application of culturally acceptable pain reduction methods are necessary to improve the menstrual wellbeing of young adolescent women with CP. Research suggests that 45 to 60 min of exercise performed three times or more per week can produce clinically significant reductions in menstrual pain [3, 4]. Future research may explore the feasibility of exercise-based interventions with young adolescent women with CP in Bangladesh.

Drawing on feminist disability theory, the present study aimed to amplify the voices of young adolescent women with CP and their mothers and to consider the role of socio-cultural factors in young women’s experiences, rather than defining ‘problems’ as within the individual. We were however, limited in several respects. Although we ensured representation in the study of young adolescent women across age, motor impairment and income, a large proportion of young women with CP were unable to participate in interviews due to perceived cognitive or communication impairments. Future research using inclusive methodologies may address this gap. Secondly, we opted to conduct group rather than individual interviews as pre-study consultation identified this to be a more acceptable method for the study location and topic. However, this approach can limit in-depth exploration of topics, and telling of personal accounts due to lack of anonymity. In particular, eliciting descriptive accounts from young adolescent women with CP was difficult. The interviewer was careful to follow participants lead on what they could or would be willing to discuss in this context. This said, mothers were generally forthcoming and appeared eager to explain their circumstances regardless of social taboo surrounding menstrual discourse. Finally, although the interviewer for this study was Bangla speaking, local dialect at times differed and some colloquial terms lacked universality. Involvement in the interviews by a local community worker was critical to bridge understanding.

## Conclusion

Young adolescent women with CP in rural Bangladesh reported a wide range of experiences and needs in learning about and navigating the materiality of menstruation alongside discourses of ‘female maturation’ and restrictive ideals of ‘independence’ and ‘quiet endurance’. Some young adolescent women with CP demonstrated adherence to these notions whereas others described exclusions and imposed restrictions. The findings of the present study provide motivation for disability services in Bangladesh to challenge the discourse of silence around menstruation and account for the menstrual needs of young adolescent women with CP and their mothers providing menstrual care. For example, orienting programs to embed value in constructs such as ‘interdependence’ will help to positively position young women as active agents in their menstrual care and contribute to the uptake of positions of power. Provision of education will also equip young women with knowledge and skills to navigate their specific menstrual needs and address issues of systemic exclusions in the absence of school attendance. Moreover, research from the global south exploring low-cost and culturally acceptable strategies to reduce menstrual pain such as exercise-based interventions are urgently needed as are interventions to alleviate distress among mothers providing menstrual care. Finally, policy responses are required to ensure that the menstrual needs of women with disability are considered within principles of inclusive development such as the availability of menstrual hygiene resources (specifically for women who are sitting and lying for long periods) and WASH solutions accessible for menstruating women with disability.

## Data Availability

The datasets generated and/or analysed during the current study are not publically available due to containing sensitive and potentially identifying patient information. This is imposed by the Asian Institute of Disability and Development (AIDD) Institutional Review Board as part of the approval for the Bangladesh Medical Research Council ethics. Researchers may contact the AIDD for limited data access related to the Bangladesh Cerebral Palsy Register at: AIDD, House # 76 & 78, Road # 14, Block B, Banani R/A, Dhaka – 1213, Bangladesh; Phone: + 88–02-55040839; Email: disabilityasia@gmail.com.

## Abbreviations

BCPR: Bangladesh cerebral palsy register
BDT: Bangladeshi Taka
CP: Cerebral palsy
GMFCS: Gross Motor Function and Classification System
IQR: Interquartile range
LMIC: Low- and middle income country
SD: Standard deviation
